# Benchmarking and optimizing microbiome-based bioinformatics workflow for non-invasive detection of intestinal tumors

**DOI:** 10.20517/mrr.2025.75

**Published:** 2025-12-01

**Authors:** Yangyang Sun, Yongxiang Huang, Ruichen Li, Junhui Zhang, Xiaoqian Fan, Xiaoquan Su

**Affiliations:** ^1^College of Computer Science and Technology, Qingdao University, Qingdao 266071, Shandong, China.; ^2^Department of Gastroenterology, Shouguang Hospital of Traditional Chinese Medicine, Weifang 262700, Shandong, China.

**Keywords:** Colorectal cancer, adenoma, machine learning, benchmarking

## Abstract

**Background: **The human gut microbiome is closely linked to disease states, offering substantial potential for novel disease detection tools based on machine learning (ML). However, variations in feature types, data preprocessing strategies, feature selection strategies, and classification algorithms can all influence the model’s predictive performance and robustness.

**Methods:** To develop an optimized and systematically evaluated workflow, we conducted a comprehensive evaluation of ML methods for classifying colorectal cancer and adenoma using 4,217 fecal samples from diverse global regions. The area under the receiver operating characteristic curve was used to quantify model performance. We benchmarked 6,468 unique analytical pipelines, defined by distinct tools, parameters, and algorithms, utilizing a dual validation strategy that included both cross-validation and leave-one-dataset-out validation.

**Results:** Our findings revealed that shotgun metagenomic (WGS) data generally outperformed 16S ribosomal RNA gene (16S) sequencing data, with features at the species-level genome bin, species, and genus levels demonstrating the greatest discriminatory power. For 16S data, Amplicon Sequence Variant-based features yielded the best disease classification performance. Furthermore, the application of specific feature selection tools, such as the Wilcoxon rank-sum test method, combined with appropriate data normalization, also optimized model performance. Finally, in the algorithm selection phase, we identified ensemble learning models (eXtreme Gradient Boosting and Random Forest) as the best-performing classifiers.

**Conclusion: **Based on the comprehensive evaluation results, we developed an optimized Microbiome-based Detection Framework (MiDx) and validated its robust generalizability on an independent dataset, offering a systematic and practical framework for future 16S and WGS-based intestinal disease detection.

## INTRODUCTION

As a leading cause of cancer-related mortality worldwide, colorectal cancer (CRC) typically originates from the adenoma (ADA)-carcinoma sequence, a process involving progressive mutational accumulation over 10-15^[1,2]^ years that accounts for about 80% of cases. Screening at the precancerous ADA stage dramatically improves the 5-year survival rate to around 90%^[3]^, thereby enhancing clinical decision-making, reducing disease incidence, and lessening the economic impact^[4,5]^. Traditional screening methods, such as colonoscopy^[6]^, are effective for early detection and can reduce CRC incidence and mortality. Despite the efficacy of conventional screening tools such as colonoscopy, their invasive nature presents challenges related to cost, risk, and patient compliance^[6-10]^. Consequently, researchers are committed to developing reliable non-invasive diagnostic alternatives.

Analysis of the gut microbiome from fecal samples presents a promising non-invasive avenue for disease detection and is currently widely used in related research^[11-13]^. A growing body of evidence indicates that the gut microbiota is fundamentally involved in both the initiation and development of CRC^[14]^. Consequently, specific microbial signatures are being identified as potential biomarkers for assessing disease susceptibility^[15]^, predicting progression^[16]^, determining patient prognosis^[17,18]^, and anticipating therapeutic response^[19,20]^. Modern high-throughput sequencing methods, such as 16S ribosomal RNA (rRNA) gene amplicon (16S) and shotgun metagenomic (WGS)^[21]^ sequencing, have been instrumental in enabling the comprehensive profiling of these complex microbial ecosystems^[22,23]^. However, the inherent complexity of microbiome data poses substantial analytical challenges, often surpassing the capabilities of conventional statistical approaches. Consequently, machine learning (ML) methods have become indispensable for managing high-dimensional, heterogeneous microbiome datasets, facilitating accurate disease classification^[23]^.

Despite the advantages offered by ML, developing robust and clinically applicable microbiome-based disease detection models remains challenging. Data heterogeneity, influenced by data preprocessing methods (such as filtering low-abundance features)^[24]^, batch effects^[25]^, feature selection strategies^[26]^, normalization techniques^[27]^, and choice of classification algorithms^[24,28]^, frequently leads to significant inconsistencies across analytical outcomes. Furthermore, external factors such as diet^[29]^, medication use^[30,31]^, and regional disparities^[32]^ considerably influence gut microbiota composition, complicating the identification of universally consistent microbial biomarkers and limiting their practical clinical utility^[33]^. Consequently, extensive, multi-cohort systematic analyses and optimized bioinformatics workflows for CRC detection are urgently needed. In response to these challenges, Li *et al.* conducted a comprehensive evaluation of 156 unique analytical pipelines. Their work emphasized the importance of systematic evaluation^[34]^. However, systematic ML optimization strategies specifically targeting CRC and ADA disease are still relatively scarce.

This study aims to establish a standardized and systematically evaluated workflow for intestinal diseases by systematically evaluating ML methodologies applied to both WGS and 16S sequencing data, specifically targeting CRC and ADA detection. Leveraging a substantial dataset of 4,217 stool samples from multiple global cohorts, we systematically explored 15 distinct filtering thresholds, 3 batch effect correction strategies, 7 feature selection strategies, 7 normalization methods, and 6 classification algorithms. A total of 6,468 methodological combinations were rigorously evaluated to optimize accuracy and generalizability. By integrating optimal solutions derived from each analytical step, we developed a comprehensive, robust disease detection workflow, demonstrating its efficacy and reliability through validation on an independent cohort. This work establishes a rigorous technical framework for future clinical applications in intestinal disease detection.

## METHODS

### Public dataset collection

We conducted a literature search in the PubMed database with the terms “metagenomics” and “human gut mycobiome” to find publications that reported data on patients with CRC and ADA. The raw FASTQ files corresponding to the identifiers in Supplementary Table 1 were obtained from the Sequence Read Archive (SRA) and European Nucleotide Archive (ENA)^[35]^ using the SRA toolkit (v.2.9.1). The predictive modeling workflow of this study is based on metagenomic data from Sun *et al.*^[36]^, wherein taxonomic and functional profiles were generated by MetaPhlAn v.4.0.6 (database: mpa_vOct22_CHOCOPhlAnSGB_202212) and HUMAnN v.3.7 (database: UniRef90s v201901b, with default parameters), respectively.

### Sequence preprocessing and profiling

For all 16S rRNA sequencing data, we used Parallel-Meta Suite^[37]^ (PMS, v.3.73) based on the Greengenes2 database, with a 99% similarity threshold, and applied copy number correction to obtain taxonomic and functional profiles.

### Microbiome-based Detection Framework

We developed MiDx (Microbiome-based Detection Framework), a comprehensive computational framework designed to provide an end-to-end, reproducible solution for microbiome-based disease detection. The framework systematically operationalizes the modeling workflow into a sequence of core modules designed to take raw data through to a fully validated predictive model. To comprehensively benchmark our models, we evaluated a total of 6,468 unique analytical pipeline combinations. The first stage (420 combinations) focused on identifying the optimal feature representations [species-level genome bins (SGBs) or Amplicon Sequence Variants (ASVs)] and filtering strategies. The 2nd stage (6,048 combinations) then used these findings to comprehensively evaluate core downstream modules, including feature selection, data normalization, and classification algorithms. The detailed methodology is as follows.

#### Feature filtering

To assess the impact of removing low-signal features, we evaluated multiple filtering strategies implemented via the *filter.features* function in the SIAMCAT R package^[38]^. We systematically compared performance across various thresholds:

(1) Abundance: 0.001%, 0.005%, 0.01%, 0.05%, 0.1%, 0.5%, and 1%.

(2) Prevalence: 10%, 20%, and 30%.

(3) Variance: 0.0001%, 0.001%, 0.01%, and 0.1%.

(4) Pass: A baseline option that bypasses filtering and retains all features for downstream analysis.

#### Batch effect correction

To account for non-biological variability between different study cohorts, we benchmarked three distinct batch effect correction methods:

(1) The *removeBatchEffect* function (limma R package)^[39]^.

(2) The *adjust_batch* function (MMUPHin R package)^[40]^.

(3) The *ComBat* function (sva R package)^[41]^.

#### Feature selection

To establish a performance baseline, we first included a model utilizing “All features”, where no feature selection algorithm was applied. Alongside this baseline, we employed six different algorithms to identify the most discriminative microbial markers. Table 1 specifies the R package, version, and statistical thresholds applied for each approach.

**Table 1:** Feature selection strategies

**Method**	**Version / Implementation**	**Distribution**	**Criteria (CRC)**	**Criteria (ADA)**
*AncomBC* ^[42]^	AncomBC (v.2.1)	Non-parametric	*q-value *< 0.05	*q-value* < 0.5
*MetagenomeSeq* ^[43]^	metagenomeSeq (v.1.36.0)	Zero-inflated (log-) Normal	*q-value *< 0.05	*q-value* < 0. 5
*T-test*	Stats (v.4.1.3)	Normal (default)	*q-value* < 0.05	*q-value *< 0.5
*Wilcoxon rank-sum test*	Stats (v.4.1.3)	Non-parametric	*q-value *< 0.05	*q-value *< 0.5
*MaAsLin2* ^[44]^	MaAsLin2 (v.1.8.0)	Normal (default)	*q-value* < 0.05	*q-value *< 0.5
*Lefse* ^[45]^	Microeco package (v.1.4.0)	Non-parametric	*q-value* < 0.05 & LDA > 2	*q-value* < 0.5 & LDA > 2

*Lefse*: Linear discriminant analysis effect size; LDA: linear discriminant analysis.

#### Data normalization

To address the compositional nature of microbiome data, we tested six normalization methods available via the *normalize.features* function in SIAMCAT^[38]^. The methods are defined as follows:

(1) Rank.unit: This method first converts all feature abundances to ranks. Normalization is then applied to each feature column by dividing its values by the square root of the sum of its ranks.

(2) Log.clr: Applies centered log-ratio transformation after adding pseudo-counts.

(3) Log.std: Applies a log transformation to the data (after adding a pseudo-count), followed by z-score standardization.

(4) Log.unit: Applies a log transformation (after adding a pseudo-count), followed by unit-based normalization across either features or samples.

(5) Std: Applies z-score normalization directly to the feature data, without a preceding log transformation.

(6) Pass: A control option that skips normalization and passes the data to the next step without transformation.

#### Model construction

We implemented all ML models for status classification, including Random Forest (RF), eXtreme Gradient Boosting (XGBoost; XGB), multilayer perceptron (MLP), k-nearest neighbors (KNN), Lasso, and support vector machine (SVM), leveraging the scikit-learn library (v.1.3.2) within the Python 3.9 environment.

#### Model evaluation

To comprehensively assess model performance and generalizability, MiDx supports flexible validation strategies, including five-fold cross-validation and the more stringent Leave-One-Dataset-Out (LODO) cross-validation. Finally, the framework automatically computes and outputs the key performance metric - the Area Under the Receiver Operating Characteristic Curve (AUC) - to provide a quantitative and reliable basis for model selection and comparison.

#### Hyperparameter Optimization

The hyperparameters [Table 2] for each ML model were systematically optimized using Bayesian Optimization^[46]^, leveraging the implementation from the scikit-learn library.

**Table 2:** Parameters of model tuning

**Method**	**Parameter**	**Value**
RF	n_estimators (number of trees)	Integer, 100-1,000
	max_features (fraction of features)	Float, 0.1-0.9
	max_samples (fraction of samples for bootstrap)	Float, 0.1-0.9
	max_depth (maximum tree depth)	Integer, 1-10
XGBoost	n_estimators (number of boosting rounds)	Integer, 100-1,000
	max_depth (maximum tree depth)	Integer, 1-10
	learning_rate (learning rate)	Float, 0.01-0.3
	subsample (fraction of training samples)	Float, 0.2-1.0
	colsample_bytree (fraction of features)	Float, 0.2-1.0
SVM	C (regularization parameter)	Float, 10^-3^-10^3^
	gamma (kernel coefficient)	Float, 10^-3^-10^3^
LR	C (inverse of regularization strength)	Float, 10^-4^-10^2^
KNN	n_neighbors (number of neighbors)	Integer, 1-50
	p (power parameter for minkowski metric)	Integer, {1, 2}
MLP	hidden_layer_sizes (network architecture)	{(32), (64), (32, 32), (64, 32)}
	alpha (L2 penalty)	Float, 10^-5^-10^-1^
	learning_rate_init (initial learning rate)	Float, 10^-5^-10^-1^
	max_iter (maximum iterations)	Integer, 100-1,000

RF: Random Forest; XGBoost: eXtreme Gradient Boosting; SVM: support vector machine; LR: logistic regression; KNN: k-nearest neighbors; MLP: multilayer perceptron.

### Statistics and bioinformatics methods

The entire analytical workflow, encompassing data processing, statistical analysis, and visualization, was implemented in the R statistical environment (v.4.4.1)^[47]^. To assess statistical differences between two groups, the Wilcoxon rank-sum test was employed for independent samples, while appropriate paired tests were used for paired observations.

## RESULTS

### Study overview and framework for a large-scale machine learning benchmark

**Figure 1:**
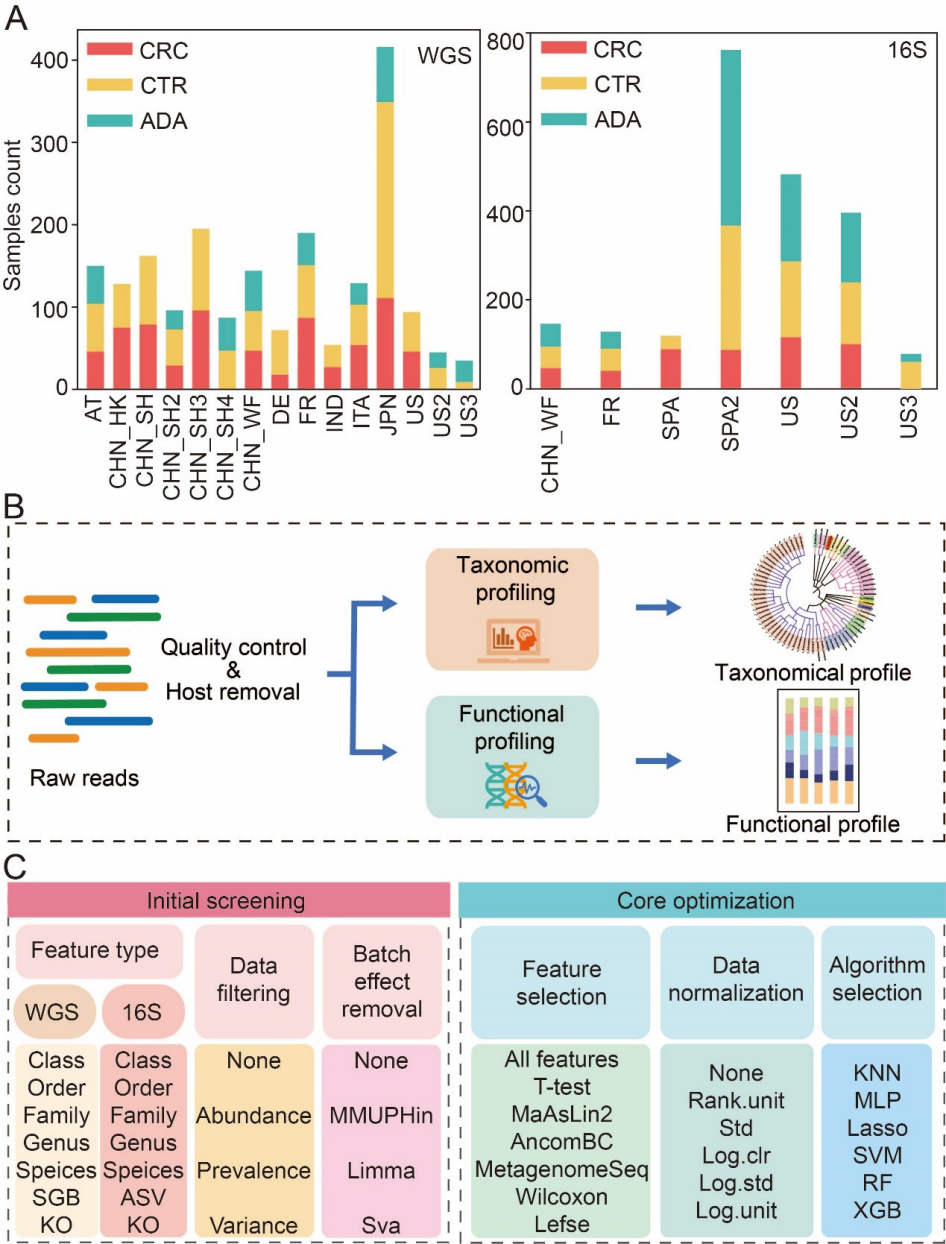
Data collection statistics and benchmark process. (A) Summary of sample aggregation and distribution; (B) A standardized bioinformatics pipeline for processing raw metagenomic data to achieve cross-cohort data harmonization; (C) Two-stage benchmarking workflow. The initial screening phase (left) evaluates various feature types, filtering methods, and batch correction strategies to identify optimal feature types and promising preprocessing strategies. Building on these findings, the core optimization phase (right) systematically evaluates combinatorial pipelines of core modules, including feature selection, data normalization, and classification algorithms. CRC: Colorectal cancer; CTR: control; ADA: adenoma; WGS: whole genome sequencing; 16S: 16S rRNA gene sequencing; AT: Austria; CHN: China; DE: Germany; ITA: Italy; JPN: Japan; IND: India; FR: France; SPA: Spain; US: United States; ASV: amplicon sequence variant; SGB: species-level genome bin; KO: Kyoto Encyclopedia of Genes and Genomes Orthology; MMUPHin: meta-analysis methods with uniform pipeline for heterogeneity integration; Limma: linear models for microarray data; SVA: surrogate variable analysis; MaAsLin2: multivariable association with linear models 2; AncomBC: analysis of composition of microbiomes with bias correction; MetagenomeSeq: MetagenomeSeq (Zero-inflated Gaussian model toolkit); Lefse: linear discriminant analysis effect size; Std: standardization; Log.clr: log centered log-ratio transformation; Log.std: log transformation with standardization; Log.unit: log transformation with unit scaling; Rank.unit: rank normalization with unit scaling; KNN: k-nearest neighbors; MLP: multilayer perceptron; Lasso: least absolute shrinkage and selection operator; SVM: support vector machine; RF: random forest; XGB: eXtreme Gradient Boosting.

To systematically explore the optimal strategy for constructing intestinal flora-based CRC and ADA detection models, we aggregated and analyzed a large-scale dataset. This dataset comprised 2,053 shotgun metagenomes and 2,164 16S rRNA gene sequencing samples sourced from 15 and 7 previously published cohorts, respectively [Figure 1A, Supplementary Tables 1 and 2]. This multi-cohort dataset spanned three health conditions: CRC, ADA, and healthy controls. A critical inclusion criterion was the collection of all samples prior to any therapeutic intervention to prevent treatment-associated biases^[20,48,49]^. Following initial quality filtering and host genome removal^[50]^, taxonomic and functional profiles for WGS data were generated using MetaPhlan4^[51]^ and HUMAnN3^[52]^ under consistent settings (refer to METHODS, Figure 1B). The 16S sequencing data were processed with the PMS^[37]^ to produce ASVs, which were then taxonomically classified against the Greengenes2 database^[53]^ (99% similarity).

To comprehensively assess the impact of various modeling steps and parameter selections on the model’s predictive performance, we broke down the model-building pipeline into the following key steps. To determine the optimal feature resolution, we evaluated model performance across seven feature types for both 16S and WGS data, spanning taxonomic ranks (Class, Order, Family, Genus, Species), functional Kyoto Encyclopedia of Genes and Genomes (KEGG) Orthologs (KOs), and fine-scale units (ASVs and SGBs) [Figure 1C]. Second, we systematically compared a range of data preprocessing strategies, including different filtering thresholds for low-abundance taxa and three widely used batch effect correction techniques. Subsequently, we used an integrated computational framework called MiDx (refer to METHODS for details) to enable systematic discovery of biomarkers and comprehensive optimization of models. Within MiDx, we benchmarked seven feature selection strategies to identify the most informative microbial signatures. These signatures were then subjected to six distinct normalization techniques to mitigate compositional bias and heteroscedasticity. Finally, the resulting feature sets were used to train and evaluate six classification algorithms. The entire analytical framework comprised 6,484 distinct tool-parameter-algorithm combinations. Crucially, the generalizability of the resulting model was rigorously confirmed using an independent validation dataset from external cohorts, underscoring its robustness across diverse conditions [Supplementary Tables 1A and 2A].

### Appropriate feature types and filtering methods improve model performance

To construct a robust and efficient ML model based on microbiome data, we performed a systematic optimization of the data preprocessing pipeline. The initial stage of this process focused on identifying the feature types that yield the highest predictive performance. To this end, we evaluated seven distinct feature types derived from human gut microbiome profiles generated via WGS and 16S sequencing. These types encompassed six levels of taxonomic resolution (Class, Order, Family, Genus, Species, and SGB or ASV), and one functional profiling approach using KEGG Orthology (KO) groups. We employed a RF classifier to benchmark the predictive power of each feature type. Model performance was rigorously assessed using the AUC metric, under both a five-fold cross-validation scheme and a more stringent LODO external validation to test for generalizability. Concurrently, this same methodological framework was utilized to explore and identify the optimal filtering threshold for low-abundance taxa, a critical step for minimizing noise and improving model fidelity.

**Figure 2:**
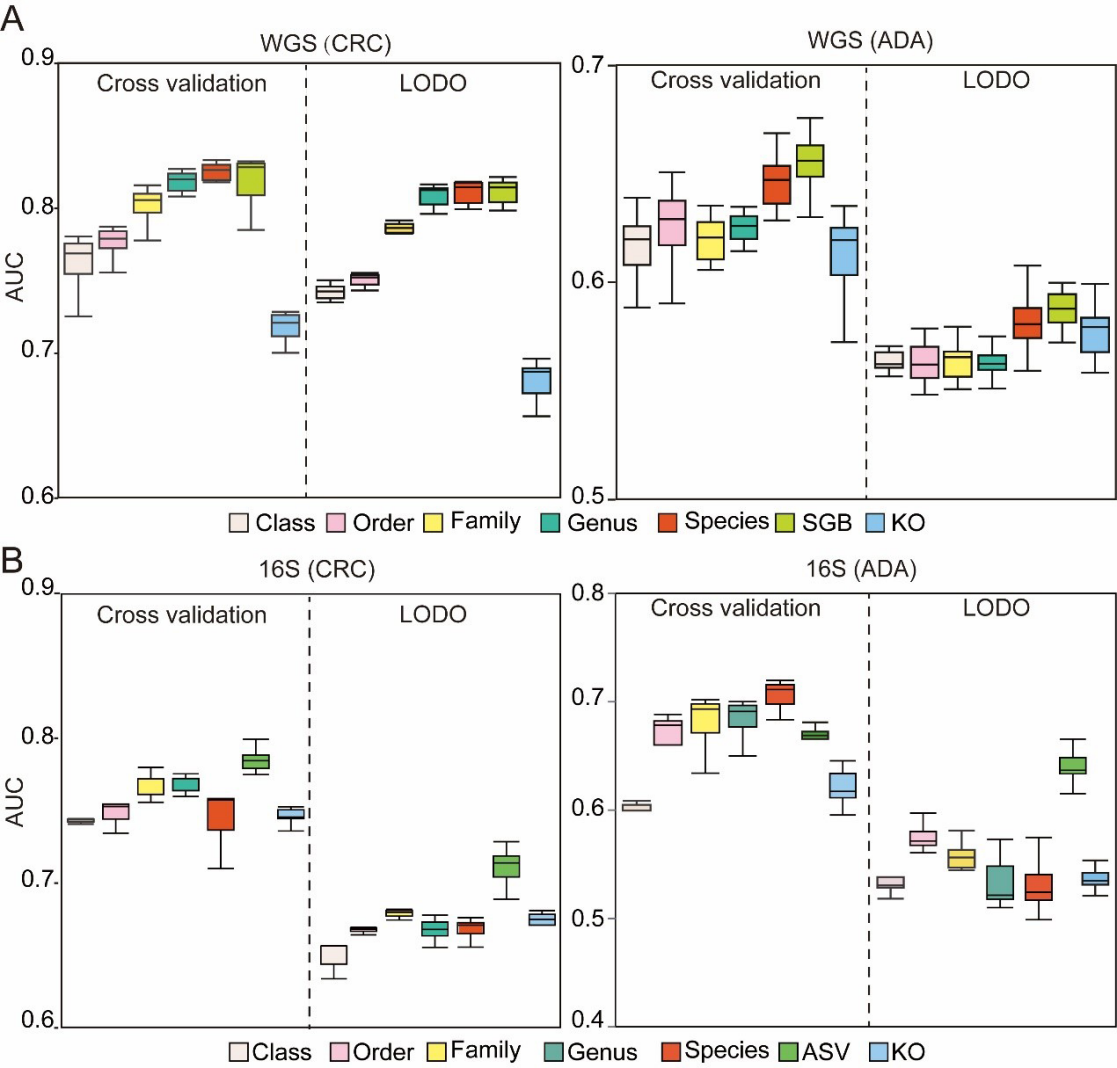
Assessment of microbiome profile. The box plots illustrate the AUC for models developed to classify (A) CRC and (B) ADA. Each plot compares seven distinct feature types. Source data is available in Supplementary dataset. WGS: Whole genome sequencing; CRC: colorectal cancer; ADA: adenoma; LODO: Leave-One-Dataset-Out; AUC: area under the curve; SGB: species-level genome bin; KO: KEGG orthology; 16S: 16S rRNA gene sequencing; ASV: amplicon sequence variant.

A key challenge in this process is the significant variation in feature space dimensionality across different taxonomic and functional levels. To determine the optimal feature type, we therefore conducted a comprehensive analysis of model performance for each modality across 15 distinct filtering thresholds (refer to METHODS for details). The filtering strategies employed were: (1) abundance-based filtering, which removes features below a minimum relative abundance; (2) prevalence-based filtering, which removes features present in a fraction of samples below a set threshold; and (3) variance-based filtering, which removes features with variance below a set threshold. By evaluating the AUC for each feature type under these varied filtering conditions, we observed clear performance trends. For WGS data, models built using features at the SGB, species, and genus levels all demonstrated high discriminatory power for both CRC and ADA in five-fold cross-validation. Critically, SGB-level features exhibited the most robust prediction performance in LODO validation, underscoring their superior generalizability [Figure 2A]. Consequently, SGB was selected as the designated feature type for all subsequent WGS analyses. For the 16S sequencing data, ASV-level features yielded the best performance for CRC classification in five-fold cross-validation [Figure 2B]. While species-level features held a marginal advantage for ADA detection in cross-validation, ASV-level features demonstrated superior generalization capabilities in the LODO validation for ADA, achieving an average AUC of 0.63. In conclusion, by balancing classification accuracy in cross-validation with generalization performance in LODO validation, we established SGB and ASV as the optimal feature types for our WGS and 16S data, respectively. These feature sets were subsequently used for all downstream analysis. 

**Figure 3:**
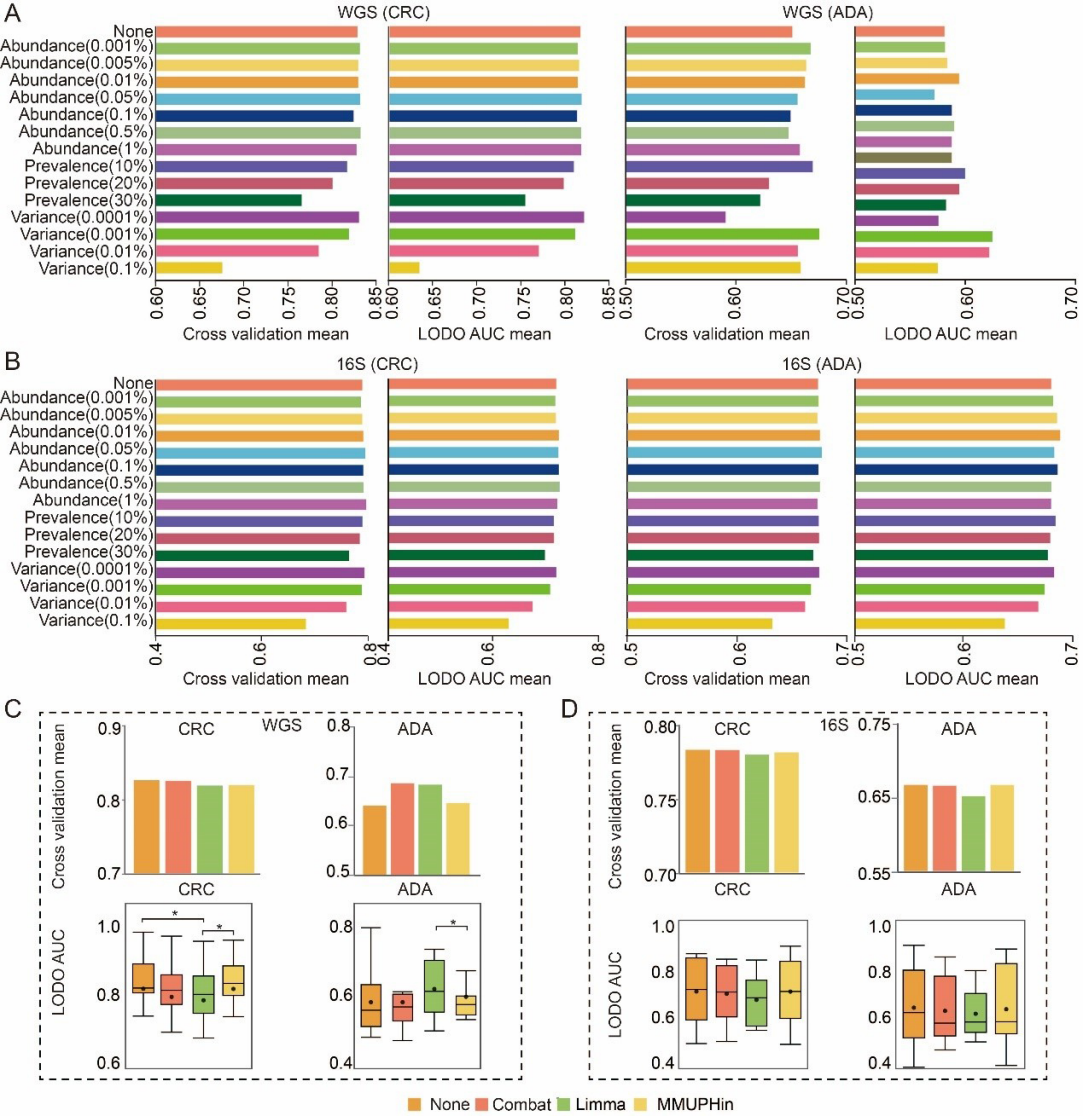
Exploration of optimal data preprocessing methods. (A and B) Comparison of 14 feature filtering thresholds against an unfiltered baseline on (A) WGS-SGB and (B) 16S-ASV data. Bars show the mean AUC from five-fold cross-validation (CV) and LODO validation; (C and D) Performance comparison between uncorrected models and three distinct batch correction methods on (C) WGS-SGB and (D) 16S-ASV data. Bar charts show the mean CV-AUC, and box plots illustrate the distribution of LODO-AUCs. Statistical significance against the baseline (uncorrected) was determined using a two-sided Wilcoxon rank-sum test (* *P-value* < 0.05). Source data is available in Supplementary dataset. WGS: whole genome sequencing; CRC: colorectal cancer; ADA: adenoma; 16S: 16S rRNA gene sequencing; AUC: area under the curve; LODO: Leave-One-Dataset-Out; Combat: ComBat (Empirical Bayes Batch Correction); Limma: linear models for microarray data; MMUPHin: meta-analysis methods with uniform pipeline for heterogeneity integration; SGB: species-level genome bin; ASV: amplicon sequence variant.

Having defined the optimal feature types, we next systematically evaluated the impact of various feature filtering strategies on model performance. For this analysis, we compared three threshold-based filtering methods (abundance, prevalence, and variance) against an unfiltered baseline model. To quantitatively assess the impact of the filtering threshold, the AUC of the model set with the filtering threshold was compared with that of the unfiltered control model using the paired Wilcoxon signed-rank test. Our comprehensive analysis revealed that, globally, the application of abundance, prevalence, or variance filtering did not yield a statistically significant improvement in model AUC compared to the unfiltered baseline [Figure 3A]. Nevertheless, we observed that specific filtering parameters conferred modest performance advantages for particular disease contexts and feature types. For WGS-based (SGB) analysis of CRC, an abundance filter (threshold 0.5%) performed best in a composite assessment of cross-validation and LODO performance [Figure 3A]. For ADA, a variance filter (threshold 0.001% and 0.01%) was optimal in the composite evaluation [Figure 3A]. For 16S-based (ASV) analysis, an abundance filter (threshold 0.05%) was most effective for CRC, while another abundance filter (threshold 0.01%) performed best for ADA in LODO validation [Figure 3B]. Based on these observed performance trends, and to ensure the exploration of the most promising analytical avenues, we selected a subset of these filtering strategies for all subsequent in-depth studies. Although statistical significance was not broadly achieved, this pragmatic selection allows for the targeted investigation of pipeline configurations that demonstrated the greatest potential for maximizing model performance. The selected methods include abundance filtering (at thresholds of 0.5%, 0.01%, 0.05%, and 0.005%) and variance filtering (at a threshold of 0.001% and 0.01%).

### Batch correction does not consistently improve ML models’ performance

When constructing disease prediction models from multi-cohort microbiome datasets, we found that cohort factors have a significant impact on microbial communities (*p-value *< 0.01, Supplementary Figures 1 and 2). Therefore, in this study, we systematically evaluated prominent batch correction algorithms: the *removeBatchEffect* function (limma package), the *ComBat* function (sva package), and the *adjust_batch* function (MMUPHin package). Within a standardized analytical framework, we assess model performance both with and without the use of these correction techniques, while keeping all other parameters identical. Model performance was comprehensively assessed using both five-fold cross-validation and LODO validation. Our results indicate that the benefits of batch correction were highly context-dependent. A notable performance improvement was observed exclusively in the ADA prediction task using WGS-derived SGB features [Figure 3C]. In this specific scenario, both the ComBat and limma-corrected models achieved a five-fold cross-validation AUC of 0.69, a marked increase from the uncorrected baseline of 0.64. However, across all other disease contexts and data types, none of the three correction methods conferred a significant performance advantage [Figure 3C and D]. Preliminary analyses indicated that batch correction did not consistently or generally improve the performance of models built on SGB or ASV features. Furthermore, such tools should be used with caution due to the potential risk of label leakage and introduction of artifacts^[54]^. Therefore, to adhere to the principle of parsimony and avoid introducing potential artifacts, this step was omitted from all subsequent analyses.

### Appropriate feature selection strategy can improve model performance

Following the determination of optimal feature types (i.e., the SGB level for WGS data and the ASV level for 16S data) and a preliminary screening of data preprocessing strategies, we utilized the MiDx (refer to METHODS for details) to systematically benchmark seven mainstream feature selection strategies. This critical step aimed to identify the most predictive biomarkers from high-dimensional microbiome data, mitigate the “curse of dimensionality,” and enhance model generalizability^[55,56]^. We consistently used AUC as the unified metric to evaluate model performance. The same systematic procedure was applied to determine the most effective data normalization techniques and classifiers.

We compared seven feature-selection methods: (1) Lefse (assesses both statistical significance and effect size in microbiome data); (2) MaAsLin2 (generalized linear mixed models); (3) AncomBC (bias correction to address compositional effects and structural zeros); (4) MetagenomeSeq (zero-inflated Gaussian mixture models for increased sensitivity); (5) T-test; (6) Wilcoxon rank-sum test; and (7) a no-feature-selection baseline. To systematically assess the impact of different feature-selection methods, we created and evaluated a total of 1,512 composite ML pipelines by combining seven feature-selection methods with six filtering thresholds, six normalization methods, and six classification algorithms. To further validate our findings, we calculated the mean rank of each method and found a strong inverse relationship with the corresponding pipeline AUC scores. This concordance confirms that both evaluation strategies led to consistent conclusions. As a final step, we determined the prevalence of each feature-selection method within the top 1% of high-performing pipelines to identify the most consistently successful approaches. We further evaluated the mean ranking of each method across these pipelines and observed a strong inverse correlation between the average rank of a method and the AUC scores of the pipelines, indicating that the two evaluation strategies yielded consistent conclusions. Finally, we evaluated each contribution to the top 1% of high-performing composite pipelines to identify the most consistently successful methods.

**Figure 4:**
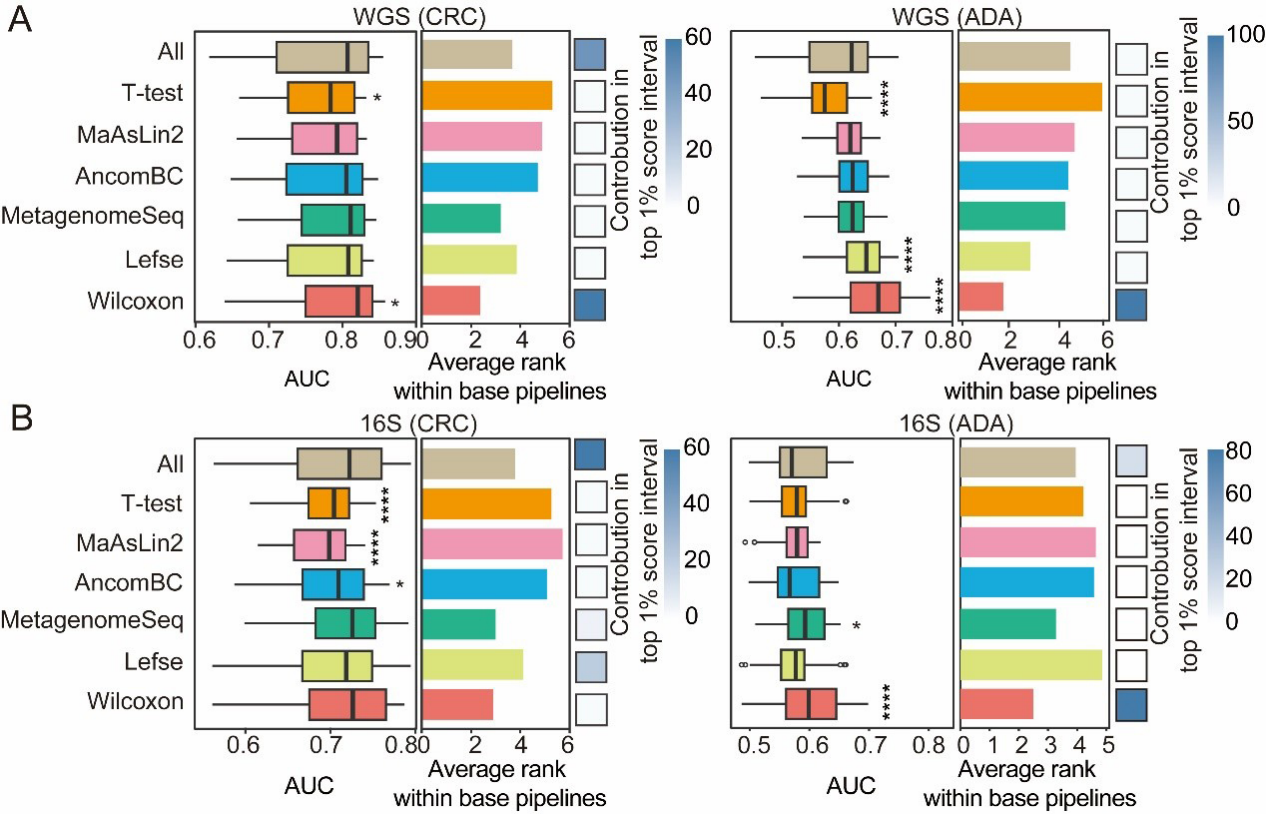
Performance comparison of feature selection strategies. For both CRC and ADA detection based on (A) WGS and (B) 16S data, this figure evaluates seven feature selection strategies across 1,512 base pipelines. The left and middle panels respectively plot the distribution of AUC scores and the average performance ranking for each strategy. The contribution of each strategy to the top 1% of processes is shown in the heat map in the right panel. Fisher’s exact test was employed to calculate the significance of this enrichment (*P-values* are indicated as follows: NS > 0.05; * < 0.05; and **** < 0.0001). Source data is available in Supplementary dataset. WGS: Whole genome sequencing; CRC: colorectal cancer; ADA: adenoma; 16S: 16S rRNA gene sequencing; AUC: area under the curve; MaAsLin2: multivariable association with linear models 2; AncomBC: analysis of composition of microbiomes with bias correction; MetagenomeSeq: MetagenomeSeq (zero-inflated Gaussian model toolkit); Lefse: linear discriminant analysis effect size.

Our results demonstrate that, for both CRC and ADA prediction, the Wilcoxon rank-sum test and MetagenomeSeq outperform the baseline across both 16S and WGS datasets [Figure 4A and B]. Specifically, Lefse dominated the top 1% of pipelines on 16S-based CRC detection, whereas Wilcoxon contributed more broadly to top-performing pipelines in other settings. In WGS-based ADA identification, Wilcoxon, Lefse, MetagenomeSeq, and AncomBC all surpassed the baseline in prediction accuracy. These findings underscore the importance of selecting an appropriate feature-selection strategy to achieve both optimal predictive performance and biological interpretability in microbiome-based detection modeling.

### Compositional data transformations markedly improve predictive accuracy

Microbiome data present inherent challenges for model training and interpretation^[56]^. First, they are compositional: sequencing yields only relative abundances rather than absolute counts, so the sum of all features within each sample is constrained to a constant. Second, these datasets are highly sparse, with zeros that may represent true biological absence (structural zeros) or artifacts of limited sequencing depth or technical noise (pseudo-zeros)^[42]^. Consequently, properly addressing these data characteristics by selecting and applying suitable normalization and transformation methods is a critical prerequisite for any subsequent analysis.

**Figure 5:**
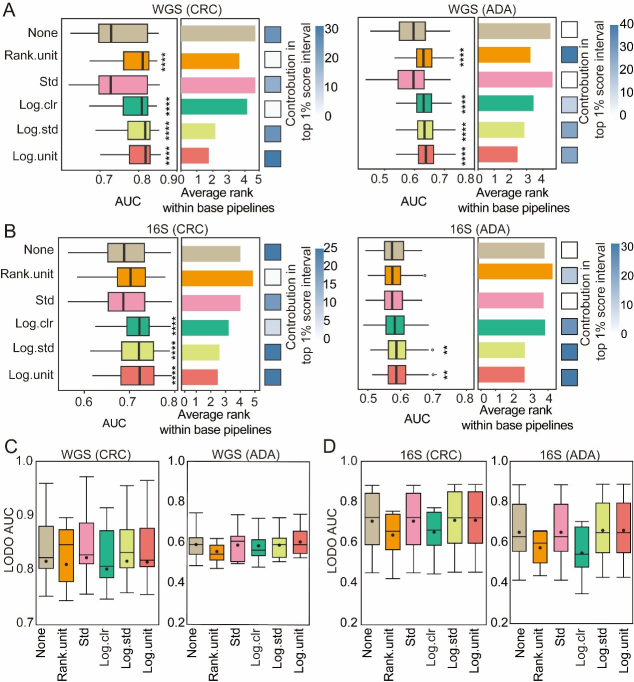
Evaluation of normalization methods. For both CRC and ADA detection based on (A) WGS and (B) 16S data, this figure evaluates 6 normalization methods across 1,512 base pipelines. The left and middle panels respectively plot the distribution of AUC scores and the average performance ranking for each strategy. The contribution of each strategy to the top 1% of processes is shown in the heat map in the right panel. Fisher’s exact test was employed to calculate the significance of this enrichment (*P-values* are indicated as follows: NS > 0.05; * < 0.05; ** < 0.01; and **** < 0.001); (C and D) Performance in LODO validation. Box plots show the distribution of LODO-AUCs for the six normalization methods using (C) WGS and (D) 16S data. Source data is available in Supplementary dataset. WGS: Whole genome sequencing; CRC: colorectal cancer; ADA: adenoma; 16S: 16S rRNA gene sequencing; AUC: area under the curve; LODO: Leave-One-Dataset-Out; Std: standardization; Log.clr: log centered log-ratio transformation; Log.std: log transformation with standardization; Log.unit: log transformation with unit scaling; Rank.unit: rank normalization with unit scaling.

We systematically evaluated six preprocessing schemes - no processing (pass), standard‐deviation scaling (std), rank unitization (rank.unit), logarithmic unitization (log.unit), logarithmic standard‐deviation scaling (log.std), and centered log‐ratio transformation (log.clr) - to determine their effects on CRC and ADA prediction performance [Figure 5A-D]. Across both WGS and 16S datasets, logarithmic and rank‐based transformations (log.unit, log.std, rank.unit, log.clr) consistently outperformed the untransformed baseline. Pipelines incorporating log.std or log.unit were especially enriched among the top 1% of performers, while for ADA detection, rank.unit and log.clr featured most prominently in that elite subset [Figure 5A and B]. Under more stringent LODO validation on WGS data [Figure 5C], certain transformations still yielded significant gains over the untransformed model: in CRC tasks, rank.unit, log.std, and std all surpassed baseline accuracy; in WGS ADA tasks, log.unit, log.std, and std showed relative advantages. In summary, logarithmic and rank‐based preprocessing methods are generally most effective at mitigating biases from compositionality and sparsity. Nevertheless, optimal choice depends on disease phenotype, sequencing modality, and specific modeling goals, and should always be confirmed through empirical evaluation to maximize predictive accuracy and cross-cohort generalizability.

### Ensemble learning classifiers demonstrate optimal performance

In the final stage, we assessed six classification algorithms, each with distinct capabilities in forming decision boundaries-hypersurfaces that divide the feature space into classes. In linear models, hyperplane decision boundaries are formed through a linear combination of the input features. This study tested two such models: logistic regression (LR) and Lasso regression. To capture the nonlinear relationship between features and categories - and thus generate more complex decision boundaries - we examined three types of nonlinear models: KNN, MLP, and radial basis function-based support vector machine (RBF-SVM). Previous studies have shown that these nonlinear models have excellent performance in predicting host phenotypes based on microbiome data^[22]^. In addition, two ensemble learning methods (RF and XGB) are evaluated, which are known to enhance predictive robustness and accuracy by aggregating multiple weak classifiers. For each disease type (CRC and ADA), we independently constructed models utilizing data derived from both 16S and WGS sequencing platforms.

**Figure 6:**
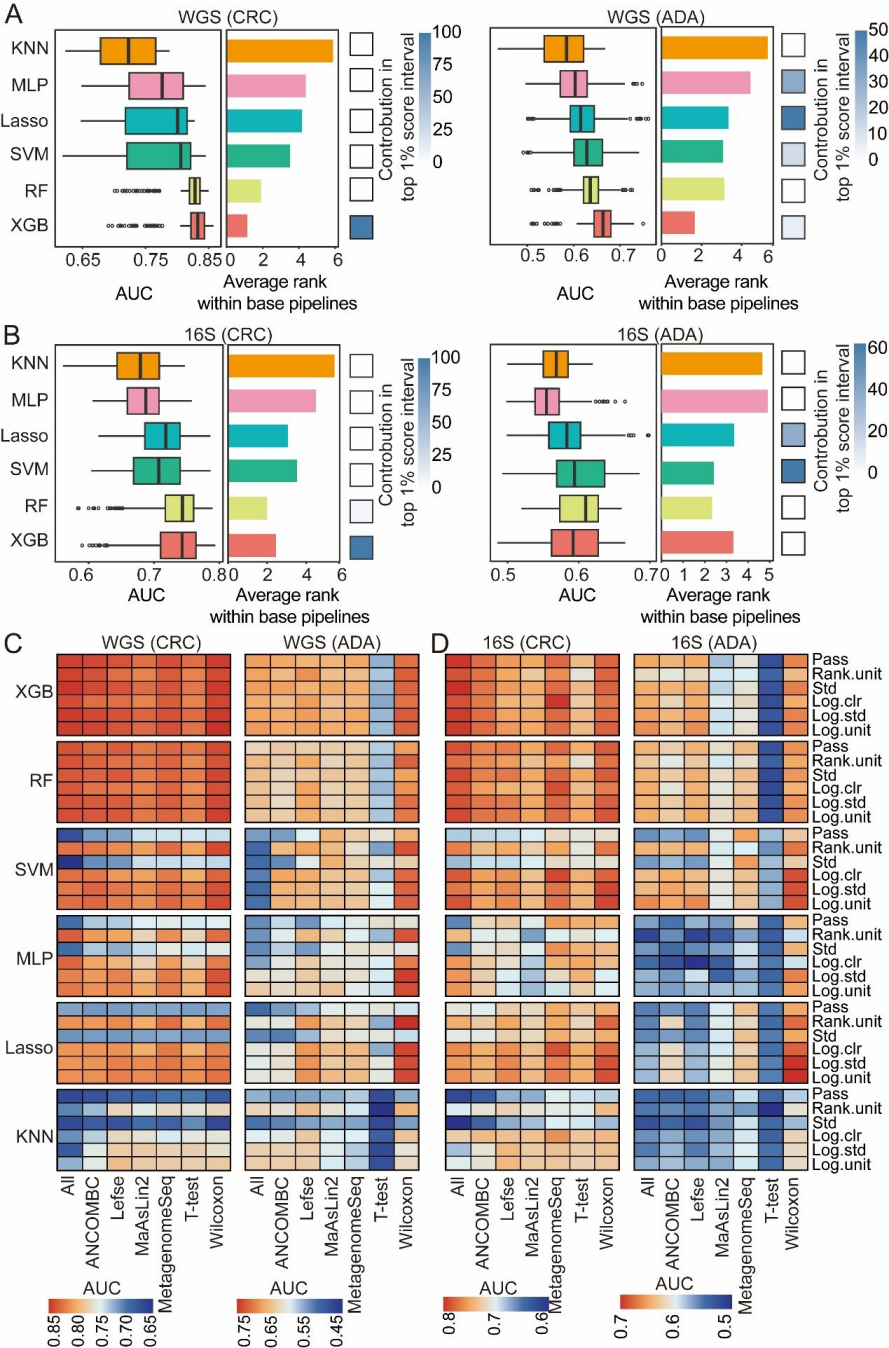
Evaluation of classification algorithms. For both CRC and ADA detection based on (A) WGS and (B) 16S data, this figure evaluates six classification algorithms across 1,512 base pipelines. The left and middle panels respectively plot the distribution of AUC scores and the average performance ranking for each strategy. The heatmaps (right panels) illustrate each strategy’s contribution to the top 1% of pipelines. The heat map shows the performance analysis results of disease detection for CRC and ADA based on WGS (C) and 16S (D) sequencing data, respectively. Source data is available in Supplementary dataset. WGS: Whole genome sequencing; CRC: colorectal cancer; ADA: adenoma; 16S: 16S rRNA gene sequencing; AUC: area under the curve; KNN: k-nearest neighbors; MLP: multilayer perceptron; Lasso: least absolute shrinkage and selection operator; SVM: support vector machine; RF: random forest; XGB: eXtreme Gradient Boosting; Std: standardization; Log.clr: log centered log-ratio transformation; Log.std: log transformation with standardization; Log.unit: log transformation with unit scaling; Rank.unit: rank normalization with unit scaling; MaAsLin2: multivariable association with linear models 2; ANCOMBC: analysis of composition of microbiomes with bias correction; MetagenomeSeq: MetagenomeSeq (zero-inflated Gaussian model toolkit); Lefse: linear discriminant analysis effect size.

In a broad assessment of performance, XGB and RF consistently outperformed all other classifiers for both disease detection tasks [Figure 6A and B]. Nevertheless, the representation of each classification algorithm within the top-tier (top 1%) of analytical processes varied significantly depending on the disease. For CRC prediction, XGB dominated the top 1% of the processes. However, in ADA prediction, in addition to XGB, Lasso, and SVM also showed good performance in the top 1% of the processes [Figure 6A and B], dominating the top 1% of high-performance ML processes. This may represent that the effective ML model types have preferences for specific diseases. In addition, we analyzed the interaction effects between model architecture, feature selection strategies, and data normalization strategies [Figure 6C and D]. XGB and RF are stable for a variety of normalization strategies due to their scale invariance and built-in feature selection. In contrast, since SVM, MLP, and Lasso have significantly reduced performance in certain normalization strategies (such as standardized std) or unnormalized data, they are more dependent on appropriate normalization. The Wilcoxon rank-sum test performed best in feature selection. Overall, the model constructed with WGS data performed better than that with 16S data [Figure 6C and D]. In summary, although nonlinear integrated models (such as XGB and RF) are generally effective tools for disease detection, the selection of the best classifier still needs to be combined with the specific disease type, data characteristics, and preprocessing strategies to maximize prediction performance.

### An optimal workflow for disease detection

**Figure 7:**
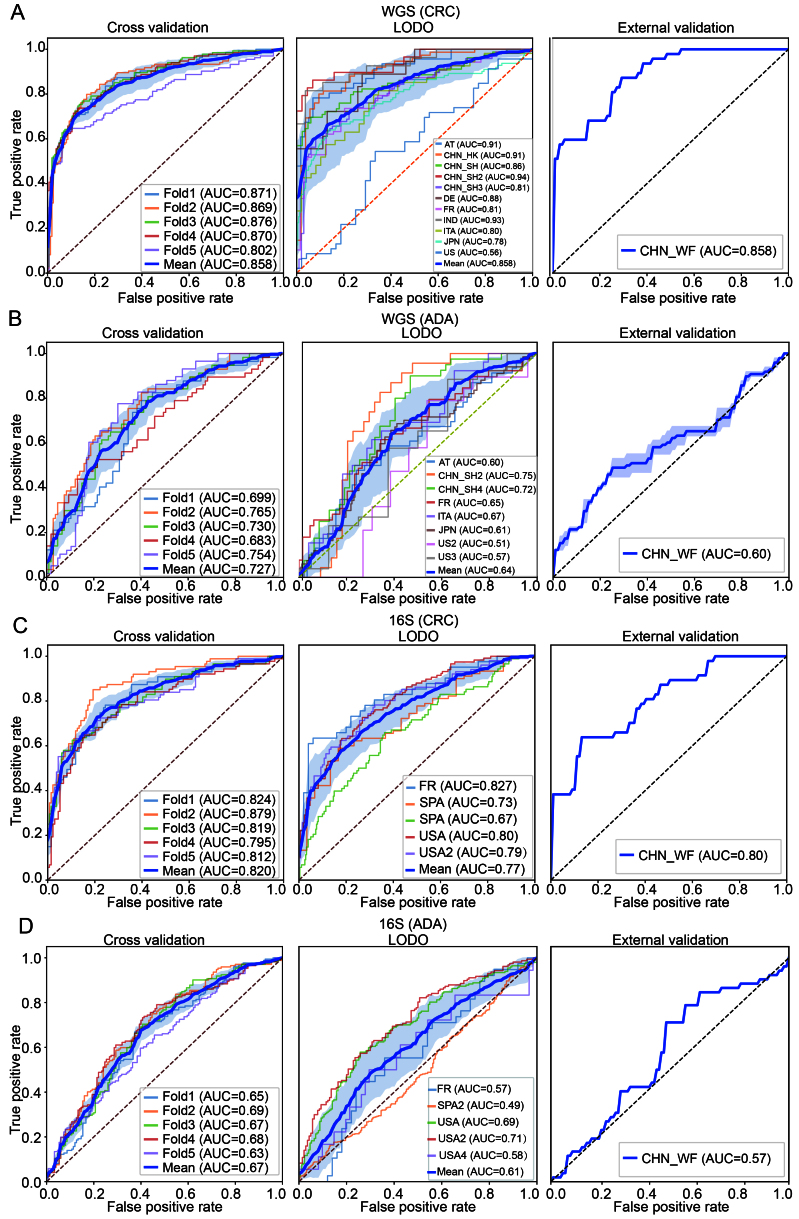
Construction and validation performance analysis of the optimal machine learning pipeline. (A and B) Classification performance evaluations of CRC and ADA based on WGS data, respectively; (C and D) Evaluation results of CRC and ADA based on 16S data, respectively. Each sub-figure shows the ROC curves under three validation strategies: five-fold cross-validation (left), LODO validation (middle), and performance on an independent validation set (right). AT: Austria; CHN: China; DE: Germany; ITA: Italy; JPN: Japan; IND: India; FR: France; SPA: Spain; US: United States; WGS: whole genome sequencing; CRC: colorectal cancer; ADA: adenoma; 16S: 16S rRNA gene sequencing; AUC: area under the curve; LODO: Leave-One-Dataset-Out.

This study systematically constructed and evaluated disease detection pipelines for CRC and ADA based on WGS and 16S data, respectively. The performance of different combinations of five modules - feature type, abundance filtering threshold, feature selection method, data transformation strategy, and classifier algorithm - was tested with five-fold cross-validation, with a total of 6,048 pipelines. We analyzed the parameter combination methods that are better adapted to different disease types and corresponding data modalities. Specifically, we selected the parameter combination for WGS-CRC detection as using a threshold of 0.5% abundance filtering, Wilcoxon rank-sum test (*q-value* < 0.05), log.unit transformation, and XGB classifier, and the average AUC of the five-fold cross-validation reached 0.85. The combination selected for WGS-ADA detection was a threshold of 0.01% abundance filtering method, Wilcoxon rank-sum test (*q-value* < 0.5), rank.unit transformation and XGB, and the average AUC of the five-fold cross-validation reached 0.73. The parameter combination selected for 16S-CRC prediction was a 0.05% abundance filtering method, all features, std transformation, and XGB, and the average AUC of the five-fold cross-validation reached 0.82. The parameter combination selected for 16S-ADA prediction was a 0.05% abundance filtering method, Wilcoxon rank-sum test (*q-value* < 0.5), std transformation, and XGB, and the average AUC of the five-fold cross-validation reached 0.67 [Figure 7A-D].

To verify the generalization ability of the pipeline selected for each feature type and disease detection task, we implemented LODO validation and external independent cohort validation. The average AUC for CRC detection in WGS data was 0.86, and the average AUC for ADA detection was 0.64. The average AUC for CRC detection in 16S data was 0.77, and 0.61 for ADA detection [Figure 7A-D]. Subsequently, we trained each model 100 times on the complete discovery cohort and calculated the average AUC on an independent external validation cohort. The results showed that WGS-based CRC detection maintained high performance with an AUC of 0.86, while WGS-based ADA detection achieved an AUC of 0.60 in the validation set. In 16S data, the AUC was 0.80 for CRC detection and 0.57 for ADA detection. Using the best-performing combination of methods in each module of the ML pipeline allowed us to build a disease detection model that is highly robust and widely applicable to CRC. However, predictive performance for ADA was poor. This difference may be because CRC is often closely associated with physiological and pathological changes in the intestine^[57]^, whereas the fecal microbiome associated with ADA is more similar to that of a healthy intestine^[58,59]^. 

Furthermore, we performed a detailed analysis of the top 20 significantly differential microbial features for both CRC and ADA [Supplementary Figure 3]. For the CRC analysis, we observed a significant enrichment of multiple features belonging to the phyla *Firmicutes A* and *Bacteroidota* across both WGS and 16S data. This finding is highly consistent with previous research^[60,61]^. Notably, similar to the findings in CRC, most of the significantly different features in ADA were also dominated by members of the phylum *Firmicutes A* and *Bacteroidetes*, suggesting a certain continuity in microbiome dysbiosis between ADA and CRC. On the other hand, we also identified distinct signals for ADA, particularly the enrichment of features from the *Proteobacteria* phylum - such as *SGB10068* in WGS data and ASV *G000431455* in 16S data - which were significantly elevated in ADA samples.

## DISCUSSION

Since the intestinal microbiome is closely related to the occurrence and development of CRC and ADA, it can be used as a potential biomarker for disease detection^[62]^. Studies have shown that the intestinal flora of CRC patients shows an obvious community imbalance, while the changes in intestinal microorganisms of ADA patients relative to healthy people are not as significant as those of CRC; however, differences in the abundance of several bacterial species related to ADA formation can still be detected^[63]^. However, because the model development workflow is a complex, multi-stage process, it is crucial to benchmark different data processing and modeling strategies and develop practical guidelines for CRC and ADA detection.

In this study, we compared microbial feature types, data filtering strategies, batch effect correction, feature selection strategies, data normalization, and ML algorithms. We used data from more than 4,000 stool samples (including 16S and WGS data) for modeling analysis. To systematically determine the optimal feature type and filtering strategy, we constructed and evaluated RF models for both CRC and ADA prediction across WGS and 16S datasets. This comprehensive benchmark involved testing 420 distinct analytical pipelines, which comprised every combination of seven feature types and fifteen filtering thresholds, with performance assessed using both cross-cohort and LODO validation. We found that for WGS data, species-level genome bins (SGB), species-level, and genus-level features had high predictive performance, among which SGB-level features showed the best generalization ability [Figure 2A]. In 16S sequencing data, ASV-level features had the best model performance [Figure 2B]. Therefore, we used SGB (WGS sequencing data) and ASV (16S sequencing data) data for subsequent analysis. Subsequently, an initial set of fifteen thresholds for the removal of low-abundance microorganisms was evaluated. This pool was then narrowed down to six promising candidates for downstream analysis [Figure 3A and B]. Furthermore, caution is warranted when correcting for batch effects to prevent overcorrection, which can inadvertently obscure or remove genuine disease-related biological signatures^[23,34]^. Our baseline analysis showed that batch correction had only a limited effect on model accuracy [Figure 3C and D], so we did not use batch correction in our subsequent analyses. 

Building upon the established optimal features (SGB or ASV) and the refined preprocessing pipeline, we utilized the MiDx (refer to METHODS for details) to systematically optimize the three core modules: feature selection, data normalization, and classifier algorithms. Specifically, we established a total of 6,048 process analyses based on six filtering thresholds, seven feature selection strategies, six data normalization methods, and six classification models. We found that the models established after the Wilcoxon rank sum test, MetagenomeSeq, and Lefse screening features were superior to those established using all features, verifying the key value of feature selection strategies in feature dimensionality reduction and improving model prediction performance [Figure 4A and B]. In addition, we found that component transformation (especially logarithm, rank, and clr transformation) can alleviate the component and sparsity of microbiome data, and improve the classification effect significantly [Figure 5A and B]. Finally, among the six classifier models, RF and XGB showed the best classification performance [Figure 6A and B]. In addition, XGB and RF performed best in various preprocessing combinations due to their automatic feature screening capabilities, insensitivity to feature scale, and robustness to high-dimensional noise^[34]^. Moreover, our systematic evaluation identified specific optimal strategies for each analytical context. For WGS data, the best-performing pipelines consistently combined the Wilcoxon rank-sum test with compositionality-aware transformations (e.g., log.unit or rank.unit), whereas for 16S ASV data, a simpler standardization (std) approach was superior. This underscores that addressing key data properties such as compositionality and sparsity is a foundational step for maximizing model performance across all microbiome sequencing modalities. Based on these findings, we recommend that in most microbiome disease detection tasks, integrated learning models such as RF or XGB are preferred, combined with effective feature selection and log/rank transformation to minimize statistical bias and improve prediction performance.

### Limitations

Despite its contributions to establishing a data-driven approach for CRC and ADA detection using fecal samples, the present study is not without limitations. First, the lack of comprehensive metadata (such as demographics, clinical characteristics, and dietary factors) may limit the biological and clinical interpretability of the microbial features selected by the model. Future studies should incorporate these metadata into the analysis to further improve the models’ predictive accuracy and generalizability. Second, because the main focus of this study was to establish the optimal computational framework, a comprehensive biological analysis of the selected biomarkers was not conducted. Although some of our selected signatures revealed microbial associations with CRC or ADA progression (e.g., members of the *Firmicutes*, and *Proteobacteria* phyla), providing additional support for the biological plausibility of our model, this is not a substitute for systematic validation. Therefore, these potential microbial markers identified by our models still need to be experimentally validated in subsequent studies to determine their etiological significance. Future in vitro and in vivo experiments are required to verify the clinical utility of these biomarkers and to further explore their therapeutic potential.

## DECLARATIONS

### Availability of data and materials

All source codes and analysis scripts developed for this study are publicly available in the GitHub repository at https://github.com/qdu-bioinfo/MiDx. This repository also contains the Supplementary Dataset (MiDx-Supplementary_dataset.xlsx) used in the analysis. The raw public datasets analyzed during the study are available in the Sequence Read Archive (SRA) and European Nucleotide Archive (ENA) databases; accession numbers are provided in Supplementary Tables 1 and 2.

### Copyright

© The Author(s) 2025.
