# Supplementary material for: Benchmarking and optimizing microbiome-based bioinformatics workflow for non-invasive detection of intestinal tumors
Source: Microbiome Res Rep. 2025 Dec 1;4(4):43. doi: 10.20517/mrr.2025.75 (PMC12702651; doi:10.20517/mrr.2025.75)
Supplement: Supplementary file 1 [file mrr-4-4-43-SupplementaryMaterials.zip › mrr4075-SupplementaryMaterials.pdf]

Supplementary Materials

Benchmarking and optimizing microbiome-based bioinformatics workflow for non-invasive detection of intestinal tumors

Yangyang Sun[1], Yongxiang Huang[1], Ruichen Li[1], Junhui Zhang[1], Xiaoqian Fan[2], Xiaoquan Su[1]

[1]College of Computer Science and Technology, Qingdao University, Qingdao 266071, Shandong, China.
[2]Shouguang Hospital of Traditional Chinese Medicine, Weifang 262700, Shandong, China.

Correspondence to: Prof. Xiaoquan Su, College of Computer Science and Technology, Qingdao University, Qingdao 266071, Shandong, China. E-mail: suxq@qdu.edu.cn; Dr. Xiaoqian Fan, Shouguang Hospital of Traditional Chinese Medicine, Weifang 262700, Shandong, China. E-mail: sgszyyyywk@wf.shandong.cn

Supplementary Table 1. Metagenome cohorts and samples
(A) Discovery dataset.

Cohort	# of CTR	# of CRC	# of ADA	Country/Region	Source
AT[1]	58	46	46	Austria	PRJEB7774
IND[2]	30	30	0	India	PRJNA531273<br>PRJNA397112
ITA[3]	51	59	27	Italy	SRP136711
JPN[4]	245	111	67	Japan	DRA008156
GE[5]	57	20	0	Germany	PRJEB27928
CHN_HK[6]	53	75	0	HK, China	PRJEB10878
CHN_SH[7]	85	80	0	China	PRJNA731589
FR[8]	66	88	40	France & Germany	ERP005534
US[9]	48	46	0	USA	PRJEB12449

CHN_SH-2[10]	44	32	23	China	PRJNA514108
CHN_SH-3[11]	100	100	0	China	PRJNA763023
US-2[12]	28	0	26	USA & Canada	PRJNA389927
US-3[13]	9	0	26	USA	PRJNA745329
CHN_SH-4[14]	47	0	40	China	PRJNA706060
CHN_WF[15,16]	48	48	54	China	PRJNA1198725

Supplementary Table 2. 16S cohorts and samples

(A) Discovery dataset.

Cohort	# of CTR	# of CRC	# of ADA	Country/Region	Source
FR[17]	50	41	38	France	PRJEB6070
US[18]	172	120	198	USA	PRJNA290926
US2[19]	141	101	162	USA	PRJNA318004
US3[20]	76	0	19	USA	PRJNA534511
SPA[21]	30	95	0	Spain	PRJNA911189
SPA2[22]	282	90	401	Spain	PRJEB71787
CHN_WF[15,16]	48	47	53	China	PRJNA1198725

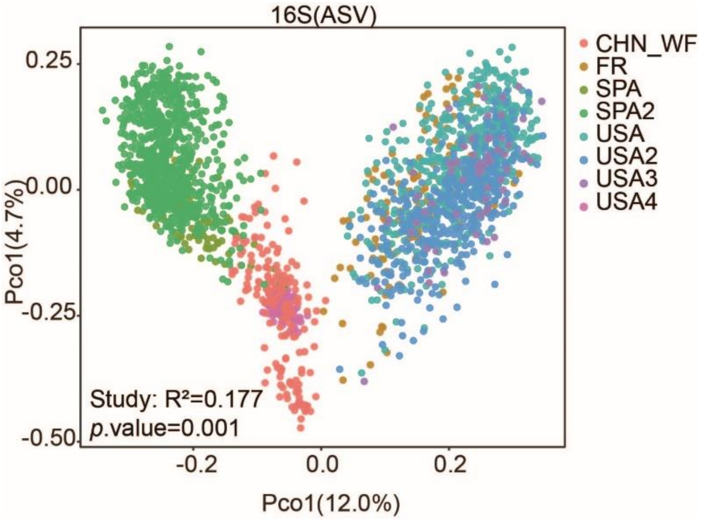

Supplementary Figure 1. Beta diversity of discovery cohorts in 16S (ASV) data. Principal coordinates analysis (PCoA) based on Bray-Curtis distances.

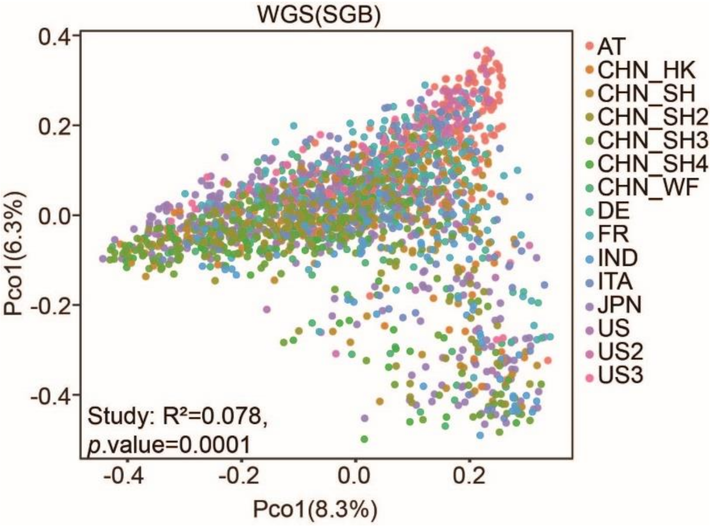

Supplementary Figure 2. Beta diversity of discovery cohorts in WGS (SGB) data. Principal coordinates analysis (PCoA) based on Bray-Curtis distances.

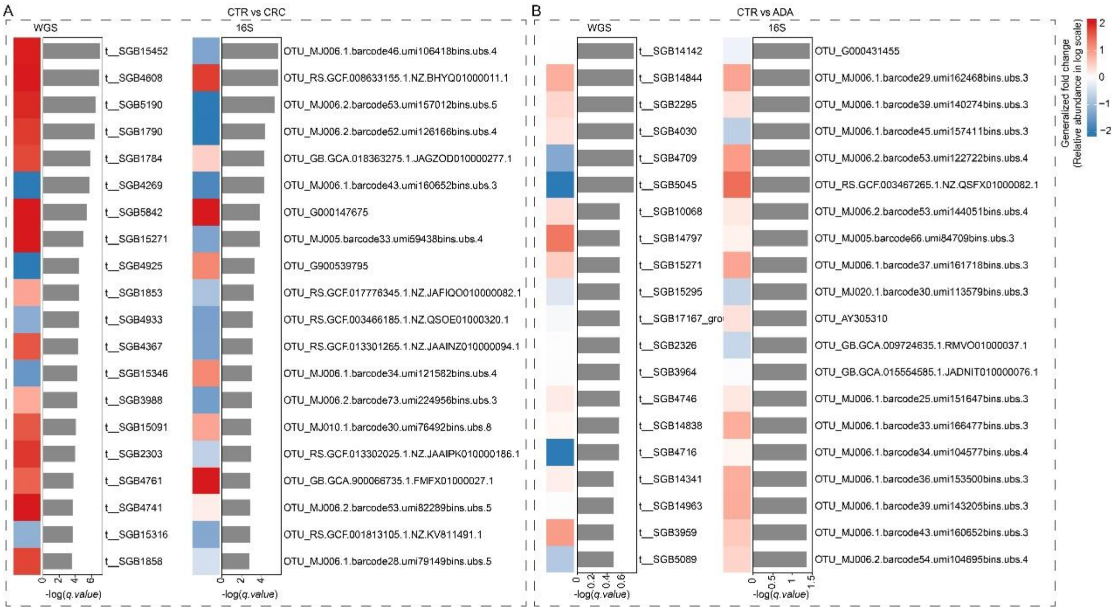

Supplementary Figure 3. Key microbial biomarkers for CRC and ADA from WGS and 16S data. (A) CTR vs. CRC; (B) CTR vs. ADA. Each panel shows two heatmaps: WGS (SGBs, left) and 16S (ASVs, right), illustrating the top 20 features ranked by *q.value*. Colors correspond to the generalized fold change, with red signifying features enriched in cases and blue signifying depletion. The accompanying grey bars indicate the -log10(*q.value*) of each feature (capped for visualization).

REFERENCES

1 Feng, Q. *et al.* Gut microbiome development along the colorectal adenoma–

carcinoma sequence. *Nature Communications* **6**, 6528 (2015).

2 Gupta, A. *et al.* Association of Flavonifractor plautii, a flavonoid-degrading bacterium, with the gut microbiome of colorectal cancer patients in India. *MSystems* **4**, 10-1128 (2019).

3 Thomas, A. M. *et al.* Metagenomic analysis of colorectal cancer datasets identifies cross-cohort microbial diagnostic signatures and a link with choline degradation. *Nature Medicine* **25**, 667-678 (2019).

4 Yachida, S. *et al.* Metagenomic and metabolomic analyses reveal distinct stage-specific phenotypes of the gut microbiota in colorectal cancer. *Nature Medicine* **25**, 968-976 (2019).

5 Wirbel, J. *et al.* Meta-analysis of fecal metagenomes reveals global microbial signatures that are specific for colorectal cancer. *Nature Medicine* **25**, 679-689 (2019).

6 Yu, J. *et al.* Metagenomic analysis of faecal microbiome as a tool towards targeted non-invasive biomarkers for colorectal cancer. *Gut* **66**, 70-78 (2017).

7 Liu, N.-N. *et al.* Multi-kingdom microbiota analyses identify bacterial–fungal interactions and biomarkers of colorectal cancer across cohorts. *Nature Microbiology* **7**, 238-250 (2022).

8 Zeller, G. *et al.* Potential of fecal microbiota for early-stage detection of colorectal cancer. *Molecular Systems Biology* **10**, 766 (2014).

9 Vogtmann, E. *et al.* Colorectal Cancer and the Human Gut Microbiome: Reproducibility with Whole-Genome Shotgun Sequencing. *PLOS ONE* **11**, e0155362 (2016).

10 Gao, R. *et al.* Integrated analysis of colorectal cancer reveals cross-cohort gut microbial signatures and associated serum metabolites. *Gastroenterology* **163**, 1024-1037. e1029 (2022).

11 Yang, Y. *et al.* Dysbiosis of human gut microbiome in young-onset colorectal cancer. *Nature Communications* **12**, 6757 (2021).

12 Hannigan, G. D., Duhaime, M. B., Ruffin IV, M. T., Koumpouras, C. C. & Schloss, P. D. Diagnostic potential and interactive dynamics of the colorectal cancer virome. *MBio* **9**, 10.1128/mbio. 02248-02218 (2018).

13 Avelar-Barragan, J. *et al.* Distinct colon mucosa microbiomes associated with tubular adenomas and serrated polyps. *npj Biofilms and Microbiomes* **8**, 69 (2022).

14 Gao, R. *et al.* Alterations, interactions, and diagnostic potential of gut bacteria and viruses in colorectal cancer. *Frontiers in cellular and infection microbiology* **11**,

657867 (2021).
15 Sun, Y. *et al.* Optimizing Metagenome Analysis for Early Detection of Colorectal Cancer: Benchmarking Bioinformatics Approaches and Advancing Cross-Cohort Prediction. *bioRxiv*, 2025.2002. 2022.639690 (2025).
16 Shen, Q., Fan, X., Sun, Y., Gao, H. & Su, X. TaxaCal: enhancing species-level profiling accuracy of 16S amplicon data. *BMC bioinformatics* **26**, 1-14 (2025).
17 Zeller, G. *et al.* Potential of fecal microbiota for early-stage detection of colorectal cancer. *Molecular Systems Biology* (2014).
18 Baxter, N. T., Koumpouras, C. C., Rogers, M. A. M., Ruffin, M. T. & Schloss, P. D. DNA from fecal immunochemical test can replace stool for detection of colonic lesions using a microbiota-based model. *Microbiome* **4** (2016).
19 Lozupone, C. & Knight, R. UniFrac: a new phylogenetic method for comparing microbial communities. *Applied and environmental microbiology* **71**, 8228-8235 (2005).
20 Dadkhah, E. *et al.* Gut microbiome identifies risk for colorectal polyps. *BMJ Open Gastroenterology* **6**, e000297 (2019).
21 Conde-Pérez, K. *et al.* The multispecies microbial cluster of Fusobacterium, Parvimonas, Bacteroides and Faecalibacterium as a precision biomarker for colorectal cancer diagnosis. *Molecular Oncology* **18**, 1093-1122 (2024).
22 Bars-Cortina, D. *et al.* Comparison between 16S rRNA and shotgun sequencing in colorectal cancer, advanced colorectal lesions, and healthy human gut microbiota. *BMC genomics* **25**, 730 (2024).
